# Prediction of thermodynamic properties of aqueous carbohydrates solution using the PHSC and ANN models

**DOI:** 10.1038/s41598-025-06552-2

**Published:** 2025-07-01

**Authors:** Soud Khalil Ibrahim, Rafid Jihad Albadr, Dharmesh Sur, Anupam Yadav, Soumya V. Menon, Debasish Shit, S. Supriya, Rajashree Panigrahi, Waam Mohammed Taher, Mariem Alwan, Mahmood Jasem Jawad, Hiba Mushtaq

**Affiliations:** 1 Department of Anesthesia Techniques, Health and Medical Techniques College, Alnoor University, Nineveh, Iraq; 2 Ahl Al Bayt University, Kerbala, Iraq; 3 Department of Chemical Engineering, Faculty of Engineering and Technology, Marwadi University Research Center, Marwadi University, Rajkot, Gujarat 360003 India; 4 Department of Computer Engineering and Application, GLA University, Mathura, 281406 India; 5 Department of Chemistry and Biochemistry, School of Sciences, JAIN (Deemed to Be University), Bangalore, Karnataka India; 6 Centre for Research Impact and Outcome, Chitkara University Institute of Engineering and Technology, Chitkara University, Rajpura, Punjab 140401 India; 7 Department of Chemistry, Sathyabama Institute of Science and Technology, Chennai, Tamil Nadu India; 8 Department of Microbiology, IMS and SUM Hospital, Siksha ‘O’ Anusandhan (Deemed to be University), Bhubaneswar, Odisha 751003 India; 9 College of Nursing, National University of Science and Technology, Nasiriyah, Dhi Qar Iraq; 10 Pharmacy College, Al-Farahidi University, Baghdad, Iraq; 11 Department of Pharmacy, Al-Zahrawi University College, Karbala, Iraq; 12 Gilgamesh Ahliya University, Baghdad, Iraq

**Keywords:** Carbohydrate, PHSC, Equation of state, Artificial neural network, Machine learning, Activity coefficient, Water activity, Chemical engineering, Chemical engineering

## Abstract

In this work the Artificial Neural Network (ANN) and the Perturbed Hard Sphere Chain (PHSC) equation of state (EoS) have been utilized to estimate the osmotic coefficient, activity coefficient, and water activity of aqueous sugar solutions containing glucose, fructose, fucose, xylose, maltose, mannitol, mannose, sorbitol, xylitol, galactose, lactose, ribose, arabinose, and sucrose. The PHSC model parameters have been adjusted using the osmotic coefficient experimental data. Then, the water activity and sugar activity coefficient were predicted. In the case of the ANN approach, six variables containing critical temperature (T_c_), critical volume (V_c_), molality, temperature, melting temperature (T_m_), and melting enthalpy (∆H_m_) of sugars have been considered as input layer. As well, 32 neurons are considered in one hidden layer. The Group Contribution (GC) method was utilized to estimate the critical properties of sugars. The training correlating coefficient (R^2^), and the Mean Square Error (MSE) have been obtained 0.999 and 2.06 × 10^–6^, respectively. The average relative deviation (ARD) value of osmotic coefficient, water activity, and sugar activity coefficient using the PHSC EoS and the ANN + GC model have been obtained 0.43%, 0.12%, 0.66%, and 2.1%, 0.89%,1.65%, respectively. The model’s performance has been evaluated using the prediction of sugar solubilities in water. The results show that the ANN + GC and PHSC model can predict the solubility data satisfactory. The ANN + GC method can be used to predict the thermodynamic properties of a new aqueous sugar solution using the molecular structure in the absence of experimental data.

**Supplementary Information:**

The online version contains supplementary material available at 10.1038/s41598-025-06552-2.

## Introduction

Sugars are composed of carbon, hydrogen, and oxygen and are found in a wide variety of foods. Sugars are known as a type of carbohydrate. Phase equilibrium calculations of sugars involve understanding the solubility of sugars in liquid solvents under varying conditions of temperature and pressure. This is crucial in fields like food science and pharmaceuticals where the stability and solubility of sugar are important. For example, understanding phase equilibrium helps in designing processes for candy making and sugar refining, ensures the stability and effectiveness of sugar-containing medications and supplements, and helps in formulating new products with desired textural and stability characteristics. In this regard, the estimation of thermodynamic properties of sugar-containing systems plays a crucial role in the phase equilibrium calculations. Estimating the thermodynamic properties of sugar-containing systems involves understanding various characteristics and behaviors of these systems, which can be complex due to interactions between solutes (sugars), solvents (usually water), and other potential components. Higher concentrations of sugar can lead to non-ideal behavior of the system. As well, interactions with acids, bases, or other solutes can alter the properties significantly. Estimating the thermodynamic properties of sugar-containing systems requires a combination of experimental data and theoretical modeling. Each specific system may present unique challenges, so a tailored approach that considers the particular sugars involved, their concentrations, and environmental conditions is crucial. For precise calculations or predictions, computational methods or simulation tools, alongside experimental validation, can provide comprehensive insights. In this work, the Artificial Neural Network (ANN) ^1^ approach and the Perturbed Hard Sphere Chain (PHSC) Equation of State (EoS) ^2–7^ have been utilized to estimate the osmotic coefficient and activity coefficient of aqueous sugar solutions over a wide range of concentrations. Thermodynamic models such as EoS and activity coefficient-based models are widely used for thermodynamic modeling of sugars-containing systems ^8–13^. Held et al. utilized the Perturbed-Chain Statistical Association Fluid Theory (PC-SAFT) EoS to correlate the osmotic coefficient of sugars in aqueous solutions ^13^. The PC-SAFT model parameters were obtained using experimental osmotic coefficient data. They predicated the solubility of sugars in water and alcohols up to high sugar concentrations. The UNIQUAC Functional-group Activity Coefficients (UNIFAC) model was used to estimate the solubility of sugars in aqueous and non-aqueous solutions ^8,12^. The osmotic coefficient (or water activity) is an important thermodynamic property in food industries because many enzymatic reactions that influence food stability are dependent on the availability of water ^14^. L. Ninni et al. estimated the water activity of various aqueous sugar solutions using the UNIFAC model ^14^. Feng et al. predicted the water activity of aqueous sugar solutions using the Statistical Association Fluid Theory (SAFT) EoS ^15^. They estimated the pure model parameters using the critical properties of sugars obtained by the Joback Group Contribution (GC) method ^15^. In this work the PHSC EoS is used to estimate the activity coefficient, and water activity of aqueous sugars solutions. The osmotic coefficient (or activity coefficient) data of sugars in water has been utilized to optimize the PHSC EoS model parameters. The sugar activity coefficient and solubility of sugars in water have been predicted without using any additional adjustable parameters. The results of the PHSC model have been compared to the ANN + GC model to evaluate the ANN performance. The ANNs have emerged as powerful tools for thermodynamic modeling due to their ability to capture complex, non-linear relationships in data. They are increasingly used to model the thermodynamic properties and behavior of systems where traditional methods may be inadequate or cumbersome. ANNs can model highly non-linear relationships between input and output variables, which is common in thermodynamic systems. As well, they rely on empirical data, making them useful when theoretical models are difficult to formulate or are too complex.

The phase behavior of mixtures, such as predicting vapor–liquid equilibrium (VLE) and liquid–liquid equilibrium (LLE), reaction rates and mechanisms in chemical processes, critical properties, enthalpies, entropies, and other thermodynamic properties for complex systems can be predicted using the ANNs. Therefore, ANN in thermodynamic modeling is a promising approach that offers flexibility and powerful predictive capabilities, especially when dealing with complex, multi-component systems. In this work, the ANN + GC approach has been used to estimate the osmotic coefficient, activity coefficient, and water activity of aqueous sugar solutions. The activity coefficient is a crucial parameter in determining how real solutions deviate from ideal behavior. In sugar solutions, these coefficients are influenced by factors such as sugar type, concentration, temperature, and intermolecular interactions. After training, ANNs can provide rapid predictions of activity coefficient (or water activity), significantly speeding up the modeling process compared to classical approaches.

In summary, this work is divided into four sections: first, the sugar’s critical temperature, pressure, and volume have been estimated using the Joback GC method, and the results of the ANN approach have been reported. Then, the PHSC EoS model parameters have been adjusted using experimental osmotic coefficient data. Then, the PHSC model results have been compared to the ANN + GC approach. Finally, the solubility of sugars in water has been predicted using the ANN + GC and PHSC models.

## Models descriptions

### The PHSC EoS

The PHSC EoS is a theoretical model used to describe the thermodynamic properties of fluids, particularly those composed of chain-like molecules. It combines the concepts of hard sphere models with perturbation theory to account for the interactions between segments of the chain molecules. In the PHSC model, molecules are considered as chains of hard spheres (segments) connected. This accounts for the shape and flexibility of real molecules ^16,17^. The perturbation theory adds corrections to the hard sphere model to account for attractive forces between molecules. It allows for more accurate predictions of thermodynamic properties. The basic idea behind the perturbed hard-sphere chain (PHSC) model is to account for the interactions in a chain of hard spheres (which represents polymeric systems) while also considering perturbations that modify the behavior of the hard spheres. The PHSC EoS is primarily an extension of the hard-sphere model. The hard sphere model, which is one of the simplest models used to describe the behavior of dense fluids, assumes that molecules behave as hard, impenetrable spheres that do not interact except for collisions. While the hard-sphere model can predict certain behaviors, it doesn’t capture the complexities of real systems, such as polymer solutions or systems with long-range interactions^2^. The PHSC EoS is widely used in various industries and academic fields where accurate thermodynamic predictions of polymeric systems, colloidal suspensions, or soft matter systems are required ^3–5,18,19^. The model assumes that all particles are spherical, which may not apply to systems with elongated or irregularly shaped particles. The perturbation terms are only valid in certain regions (e.g., dilute to moderately dense systems). For very dense systems, the assumptions behind the perturbation may break down, leading to inaccurate predictions. By modeling chain-like molecules and introducing perturbation terms, this EoS can describe complex behaviors in a variety of physical systems, including polymer solutions, colloidal dispersions, and soft matter systems. However, its accuracy and applicability depend on the assumptions of the model, such as spherical symmetry and the validity of perturbation theory. The PHSC EoS is typically expressed in terms of the compressibility factor as follows:

1 $$Z = Z^{ref} + Z^{pert} + Z^{assoc}$$

where *Z*, *Z*^*ref*^, *Z*^*pert*^, and *Z*^*assoc*^ refer to the total compressibility factor, reference, perturbation, and association, respectively. The parameters, *a(T)* and *b(T)* were defined as follows ^2^:

2 $$a_{ij} (T) = \frac{2\pi }{3}\sigma_{ij}^{3} \varepsilon_{ij} F_{a} \left( {\frac{{k_{B} T}}{{\varepsilon_{ij} }}} \right)$$

3 $$b_{ij} (T) = \frac{2\pi }{3}\sigma_{ij}^{3} F_{b} \left( {\frac{{k_{B} T}}{{\varepsilon_{ij} }}} \right)$$

where *σ* and *ε* refer to segment diameter and segment energy in the PHSC EoS. In Eqs. (2) and (3), *T* and *k*_*B*_ refer to temperature and Boltzmann constant. F_a_ and F_b_ are temperature-dependent parameters; refer to original PHSC paper ^2^. The Boublik–Mansoori–Carnahan–Starling equation is used for the radial distribution function (ɡ^hs^) of hard sphere mixtures ^20^:

4 $$g_{ij}^{hs} \left( {d_{ij}^{ + } } \right) = \frac{1}{1 - \eta } + \frac{3}{2}\frac{{\zeta_{ij} }}{{(1 - \eta )^{2} }} + \frac{1}{2}\frac{{\zeta_{ij} }}{{(1 - \eta )^{3} }}$$

where *η* is reduced density and *ζ* refers to packing fraction. ${Z}^{assoc}$ is defined based on the Statistical Association Fluid Theory (SAFT) model introduced by Chapman et al. ^21^:

5 $$Z^{assoc} = \rho \sum x_{i} \mathop \sum \limits_{{A_{i} }} \left[ {\frac{1}{{X^{{A_{i} }} }} - \frac{1}{2}} \right]\left( {\frac{{\partial X^{{A_{i} }} }}{{\partial \rho_{i} }}} \right)$$

6 $$X^{{A_{i} }} = \left[ {1 + \rho \sum x_{i} \mathop \sum \limits_{{B_{j} }} X^{{B_{j} }} {\Delta }^{{A_{i} B_{j} }} } \right]^{ - 1}$$

7 $${\Delta }^{{A_{i} B_{j} }} = g_{ij}^{hs} \left( {d_{ij}^{ + } } \right)\left[ {exp\left( {\frac{{\varepsilon^{{A_{i} B_{j} }} }}{{k_{B} T}}} \right) - 1} \right]\left( {\sigma_{ij}^{3} \kappa^{{A_{i} B_{j} }} } \right)$$

where $X^{{A_{i} }}$, ${\Delta }^{{A_{i} B_{j} }}$, $\varepsilon^{{A_{i} B_{j} }}$, and $\kappa^{{A_{i} B_{j} }}$ refer to the unbonded association fraction, association strength, association energy, and association volume, respectively. This interaction often involves hydrogen bonding or dipole–dipole interactions, where molecules can form associations that impact the overall properties of the fluid. Association interactions in SAFT provide a powerful way to predict the behavior of complex fluids. They highlight the importance of molecular interactions in determining physical properties and phase behavior. Understanding this interaction is critical in various fields, including material science (such as designing polymers with specific properties), biophysics (such as protein interactions), and chemical engineering (such as emulsions and dispersions).

Using the Eqs. (1)–(7), the water activity coefficients *a*_*w*_ and activity coefficient of component *i* are calculated by:

8 $${a}_{w}=\frac{{\varphi }_{w}}{{\varphi }_{0w}(xw\to 1)}$$

where ${\varphi }_{w}$ is the fugacity coefficient of water in the mixture, and ${\varphi }_{0w}$ stands for the fugacity coefficient of pure water. The osmotic coefficient is as follows:

9 $$\phi =\frac{1000 \text{ln}({a}_{w})}{{M}_{w}\sum_{i\ne w}{\nu }_{i}{m}_{i}}$$

with *M*_*w*_ is the molecular weight of water, *m*_*i*_ is the molality, and ${\nu }_{i}$ represents the number of species per solute. The symmetric activity coefficient of component *i* is defined as follows:

10 $${\gamma }_{i}^{symmetric }=\frac{{\varphi }_{i}(T,P,xi)}{{\varphi }_{0i}(T,P,xi\to 1)}$$

The fugacity coefficient of component *i* is calculated using the PHSC EoS. For more details refer to ^3–7,18,19^.

### The ANN models

**Fig. 1:**
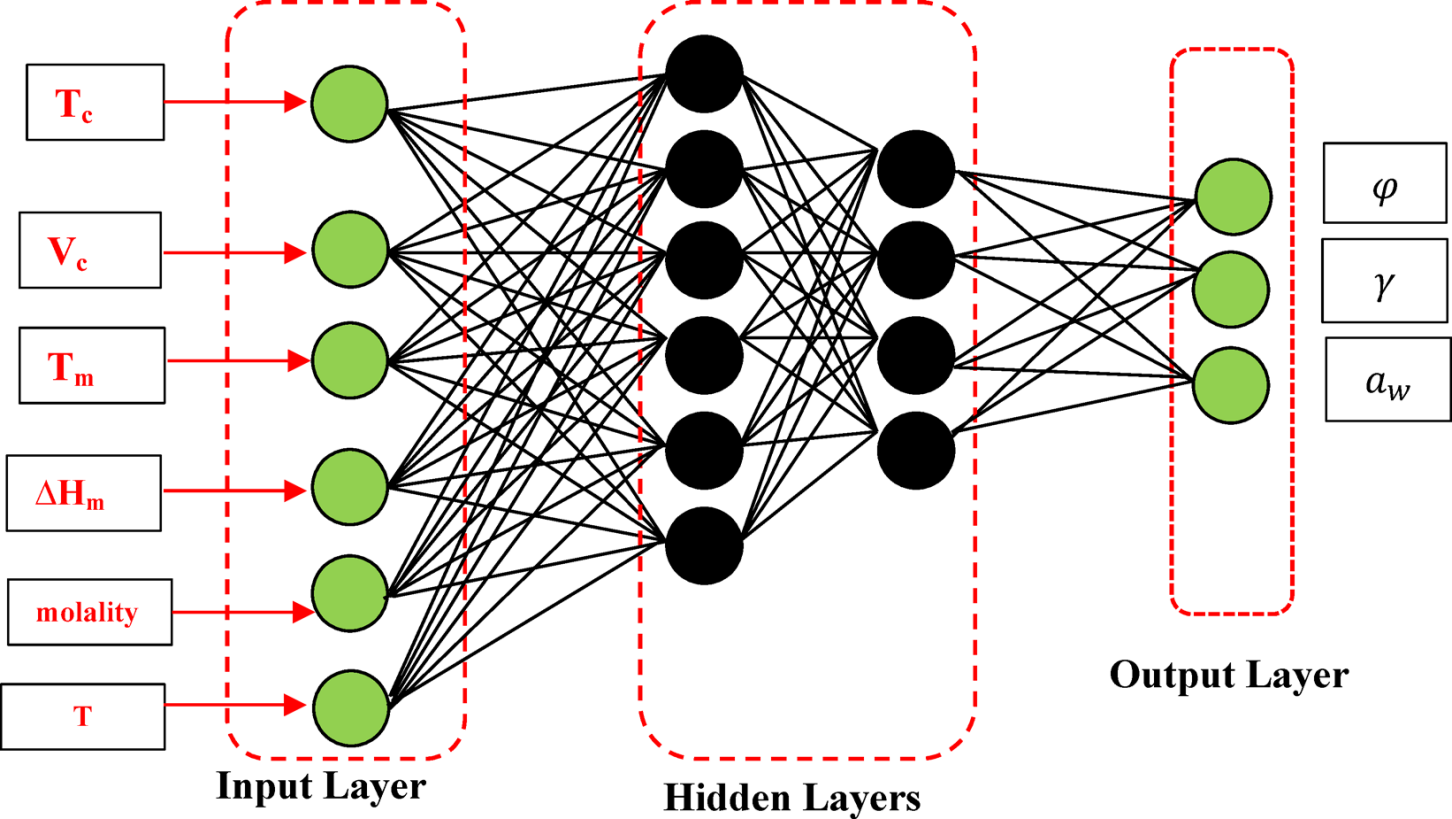
The neural network architecture diagram.

The ANN algorithm was developed based on the number of input neurons, number of hidden layers, and output neurons. The ANN models can be specified by three entities: interconnections, activation functions, and learning rules. In this work Feedforward Neural Networks (FNN) architecture has been considered. In the case of the FNN approach, the information flows in one direction from input to output and the layers are fully connected. Therefore, each neuron in a layer is connected to all the neurons in the next layer. In the ANN method, the inputs are fed to the input layer and delivered to the hidden layer using a specific transfer function. The converted inputs are sent to the output layer to estimate the desired properties. The estimated values are compared to experimental data to analyze the error using the objective function (OF). Finally, the results are fed back to the system. This process is repeated using trial and error to reach a minimum error value of OF. The number of neurons in the hidden layer in the trial and error approach is varied to reach the best OF outputs. The number of neurons in the hidden layer is obtained using the error analysis. At first, one neuron is considered to estimate the error using the training subset. Then, two neurons are considered to estimate the error. This process is continued to reach the optimum number of neurons using the minimum error value of testing subsets. Therefore, the number of neurons in the hidden layer increases if the desired error is not obtained. In this study the multilayer feed-forward network is utilized; see Fig. 1.

The neuron’s outputs in the network are estimated using the activation function and adjustable weight values. A neural network is a complex network of interconnected nodes, each representing a neuron. The neurons work together to produce output. However, not all connections between neurons are created equal. The weights are defined to determine the strength of the connections between neurons. During the training step of an ANN, the weights are adjusted iteratively to minimize the difference between the predictions and the actual target values. The weights have been optimized by using the Levenberg-Marquardt optimization algorithm ^22,23^. Weights and biases are critical concepts in ANN. These are the learnable parameters of a neural network that govern the network’s activity. The output of the ANN is more accurate by adjusting the weights and biases during training. Activation functions are used to induce non-linearity in a neuron’s output. A neuron’s output would be a linear function of its inputs without an activation function. In the next section, the results of the PHSC EoS and ANN model have been discussed.

## Results and discussion

### The ANN approach

In this study 840 data points for osmotic coefficient, activity coefficient, and water activity of aqueous sugar solutions have been collected. Data points have been divided into 600 train and 240 test/validation points (about 70% train, 15% test, and 15% validation datapoints). The melting temperature, melting enthalpy, molality, critical temperature, temperature, and critical volume of sugars have been considered as input layer. The critical properties of sugars have been estimated using the Group Contribution (GC) approach such as Joback–Reid ^24,25^. The Joback GC method is a group contribution method that estimates the critical properties (such as critical temperature and pressure) of organic compounds based on the functional groups that make up the molecule. This method uses a set of empirical correlations derived from experimental data. Sugars (carbohydrates) are generally made up of various groups such as alcohols, aldehydes, ketones, and sometimes ethers. Alcohol group (-OH) is found in the hydroxyl groups of sugars, aldehyde group (-CHO) in aldoses (like glucose), ketone group (-CO-) in ketoses (like fructose), ether group (-O-) in disaccharides and oligosaccharides.

Integrating artificial neural networks with group contribution methods like the Joback GC method presents a promising approach for enhancing the predictive capability of thermophysical properties in chemical engineering. The ANNs can capture non-linear relationships and interactions between various functional groups that traditional group contribution methods might overlook, once trained. As well, the ANN can easily adapt to new data or different classes of compounds. The model can be trained on a broader dataset that minimizes biases present in traditional group contribution table, and once established, ANNs can quickly predict properties for new compounds compared to the iterative nature of traditional methods. The ANN + GC approach can be used for new material design and chemical processes such as: assisting in designing new materials with desired thermophysical properties by predicting those values for combinations of functional groups and improving accuracy in process simulation software by providing better estimates of properties required for thermodynamic calculations. In summary, combining artificial neural networks with group contribution methods like Joback–Reid GC method ^26^ offers a powerful approach to predicting thermophysical properties. This integrated method leverages the strengths of both traditional empirical methods and modern machine learning techniques, leading to enhanced accuracy and adaptability for complex molecular systems. Using the right data and model design, this combination can significantly improve property estimation in chemical engineering and material science. In Table 1 the groups parameters of the Joback–Reid GC method have been reported.

**Table 1:** Groups considered in the Joback–Reid GC method ^26^.

Group	${tb}_{k}$	${tc}_{k}$	${vc}_{k}$
Without rings			
-CH_3_	23.58	0.0141	65
-CH_2_-	22.288	0.0189	56
> CH-	21.74	0.0164	41
>C<	18.25	0.0067	27
=CH_2_	18.18	0.0113	56
=CH-	24.96	0.0129	46
=C<	24.14	0.0117	38
-O-	22.42	0.0168	18
>C= O	94.97	0.0284	55
-OH	92.88	0.0741	28
With ring			
-CH_2_-	27.15	0.0100	48
>CH-	21.78	0.0122	38
>C<	21.32	0.0042	27

The normal boiling temperature (T_b_), critical temperature (T_c_), and critical volume (V_c_) are estimated using as follows:

11 $${T}_{b}\left(K\right)=198+\sum_{k}{N}_{k}{tb}_{k}$$

12 $${T}_{c}\left(K\right)={T}_{b}{\left[0.584+0.965\left\{\sum_{k}{N}_{k}{tc}_{k}\right\}-{\left\{\sum_{k}{N}_{k}{tb}_{k}\right\}}^{2}\right]}^{-1}$$

13 $$V_{c} \left( {\frac{{{\text{cm}}^{3} }}{{{\text{mol}}}}} \right) = 17.5 + \sum\limits_{k} {N_{k} } vc_{k}$$

where ${N}_{k}$, ${tb}_{k}$, ${tc}_{k}$, and ${vc}_{k}$ refer to number of groups, boiling temperature, critical temperature, and critical volume, respectively. In Table 2 the carbohydrates properties have been presented.

**Table 2:** Critical temperature, critical volume, and melting temperature estimated by Joback–Reid GC method.

	T_c_ (K)	V_c_ (m^3^/mol)	T_m_ (K)
Glucose	1034.02	460	423.20
Xylose	900.63	391	416.20
Arabniose	890.40	343	433.00
Xylitol	947.50	393	367.50
Ribose	900.63	391	359.00
Galactose	1034.00	460	438.15
Fructose	1017.85	415	378.20
mannose	1034.00	460	406.15
Sucrose	1782.70	784	459.15
sorbitol	1092.90	462	373.80
Maltose	1783.80	777	377.20
Mannitol	1092.90	462	439.15
Lactose	1783.80	777	498.03
Trehalose	1783.80	777	368.15
Maltitol	1658.00	601	423.00

The input layer has six neurons containing molality, critical volume, critical temperature, melting temperature, temperature, and melting enthalpy. In Table 3 (in the next sections) the melting enthalpy and melting temperature of studied sugars have been reported.

**Table 3:** The weight and Bias of hidden and output layers for 32 neurons.

	Hidden layer								Output layer		
Weights							Bias	Weight			Bias
Neurons	T_c_	V_c_	T_m_	∆H_m_	Molality	T		$\phi$	$\gamma$	${a}_{w}$	
1	-1.6033	1.9248	-0.3285	0.9153	0.7867	1.5442	2.8000	0.8348	-0.4619	0.5310	-0.2064
2	0.8398	-2.3537	-0.0946	0.7289	1.0268	0.0859	-2.6194	-0.6227	-0.4250	-0.8178	0.6170
3	0.0669	0.8562	-1.3657	-1.6789	-1.5551	-1.4916	-2.4387	0.1524	0.3667	0.0932	0.5102
4	1.8173	-0.8871	0.3439	-1.6567	-0.9421	-0.7423	-2.2581	-0.1485	0.2889	0.2952	
5	0.9064	-0.4694	-1.6658	-1.0975	-1.6789	-1.0616	-2.0774	0.3580	0.2716	0.8903	
6	2.2894	0.3649	-0.8795	0.2353	1.2794	2.3505	-1.8968	-0.5821	0.4186	-0.5275	
7	-0.2195	2.1029	0.3942	0.7336	1.6358	-0.6156	1.7161	-0.7612	0.2146	-0.0997	
8	-0.5824	-0.7085	-2.1379	-0.7958	-1.3398	0.7481	1.5355	-0.0825	0.3239	0.5406	
9	1.2127	1.8007	-0.7812	1.1928	1.0457	-0.1924	-1.3548	-0.2996	0.3240	-0.1677	
10	-2.1116	-1.0065	0.5936	1.1070	0.8889	0.8886	1.1742	0.6839	0.6658	-0.4871	
11	-1.2338	0.6764	-0.7894	1.5762	-1.6591	-0.7648	0.9935	0.2269	0.1645	0.0815	
12	-1.8254	0.9297	1.8129	0.1751	0.5710	-1.4023	0.8129	0.7399	-0.4704	-0.3639	
13	-0.5673	0.2035	2.5105	-0.9095	-0.5889	-1.4224	0.6323	-0.7616	0.8797	0.2911	
14	1.2737	0.7615	0.8850	-1.5159	1.5990	0.4882	-0.4516	-0.0411	0.2786	0.0894	
15	1.3342	0.7324	-0.6867	0.4880	-2.1940	-0.1774	-0.2710	0.2946	0.0878	0.4421	
16	-1.9886	-1.4563	0.3804	1.2614	-0.1699	-0.1961	0.0903	0.0450	0.9874	-0.5626	
17	-0.5034	-1.8589	-1.9601	-0.3825	-0.3780	1.4193	-0.0903	-0.7884	-0.7806	-0.8728	
18	0.0985	1.8289	1.4889	-1.4994	-0.1432	0.2916	0.2710	-0.1908	-0.1033	-0.2684	
19	-0.3746	-1.4808	1.7094	-1.0514	1.2164	0.1978	-0.4516	0.5270	0.2558	0.5440	
20	0.6239	-1.8592	1.2640	-1.3777	-0.7062	1.5877	0.6323	0.8657	0.9455	-0.6159	
21	0.7887	0.3772	-1.4746	-1.3497	1.7549	-0.8045	0.8129	-0.7223	0.3925	-0.8124	
22	-1.2946	2.3766	0.5872	-0.3729	-0.1783	0.6966	-0.9935	0.0508	0.0607	0.7223	
23	-0.2769	0.6855	-1.9347	0.1100	-1.8810	0.8351	-1.1742	-0.0303	-0.2131	0.3429	
24	-2.0382	-1.3022	-0.3144	-0.1791	-1.3635	-0.2437	-1.3548	0.4825	0.0401	-0.3046	
25	1.9632	-0.5316	-0.7595	1.5224	0.8994	0.2157	1.5355	-0.7000	0.1722	-0.4757	
26	-1.9520	-0.2303	-1.9853	0.1059	-0.1555	-1.1658	-1.7161	-0.9111	0.5099	-0.5144	
27	-1.2269	1.5006	-1.0009	1.3822	-1.0820	-1.1480	-1.8968	-0.1152	0.3756	-0.2815	
28	-0.8317	-2.2399	-0.5027	0.8977	-1.0357	0.1049	-2.0774	0.4727	-0.2106	0.3668	
29	-1.0883	1.2817	-1.4629	1.6501	0.3872	0.7223	-2.2581	0.4081	-0.1154	-0.9608	
30	-0.0657	0.9226	0.6279	-1.6525	-1.9645	2.5855	-2.4387	-0.3383	-0.1514	-0.4595	
31	-1.2475	-0.9616	-0.2260	1.3689	1.8531	-0.6669	-2.6194	-0.6059	0.6434	-0.1402	
32	1.8882	-1.2922	0.8192	-0.8819	-1.0751	0.1873	2.8000	0.7755	-0.2176	0.5382	

The trial and error approach is used to find the optimum ANN architecture. There are no theoretical methods to identify the best optimum ANN architecture ^27^. The results show that the optimum number of the hidden layer, and the number of neurons in the hidden layer are one and 32 respectively. As shown in Fig. 1, three outputs containing water activity, sugar activity coefficient, and osmotic coefficient have been considered in the ANN network. The ANN model performance has been evaluated using the Mean Square Error (MSE) and R^2^ as follows:

14 $$\text{MSE}\left(\text{\%}\right)=\frac{1}{N}\sum_{i=1}^{N}{{(x}_{i}^{exp}-{x}_{i}^{calc})}^{2}$$

15 $${R}^{2}=1-\frac{\sum_{i=1}^{N}{({x}_{i}^{exp}-{x}_{i}^{calc})}^{2}}{\sum_{i=1}^{N}{{(x}_{i}^{exp}-{\overline{x} }^{exp})}^{2}}$$

where ${x}_{i}^{exp}$ and ${x}_{i}^{calc}$ refer to experimental and calculated osmotic coefficient, water activity, or activity coefficient. ${\overline{x} }^{exp}$ is the average value of the experimental $x$. As well, the average absolute deviation (AAD%) of five sugars (that not considered previously for the training subset) is calculated as follows:

16 $$\text{ARD}(\text{\%})=\frac{100}{N}\sum_{i=1}^{N}\left|\frac{{x}_{i}^{exp}-{x}_{i}^{calc}}{{x}_{i}^{exp}}\right|$$

**Fig. 2:**
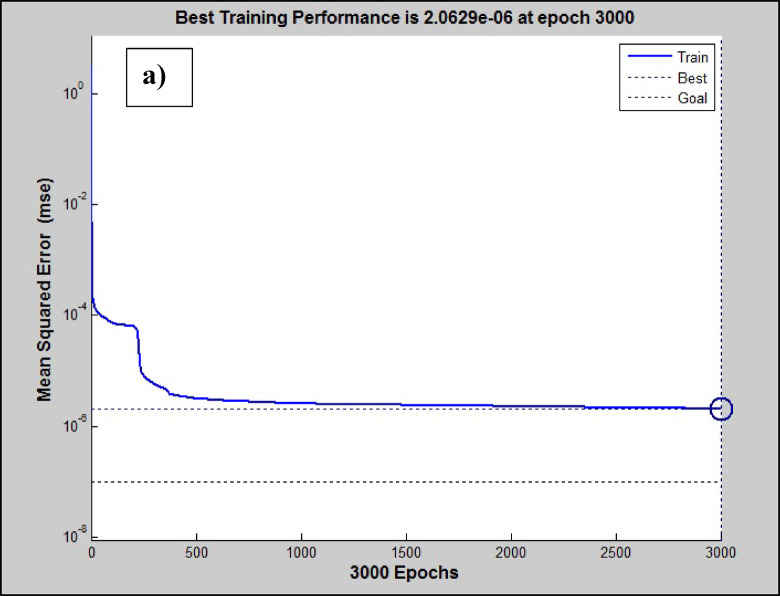

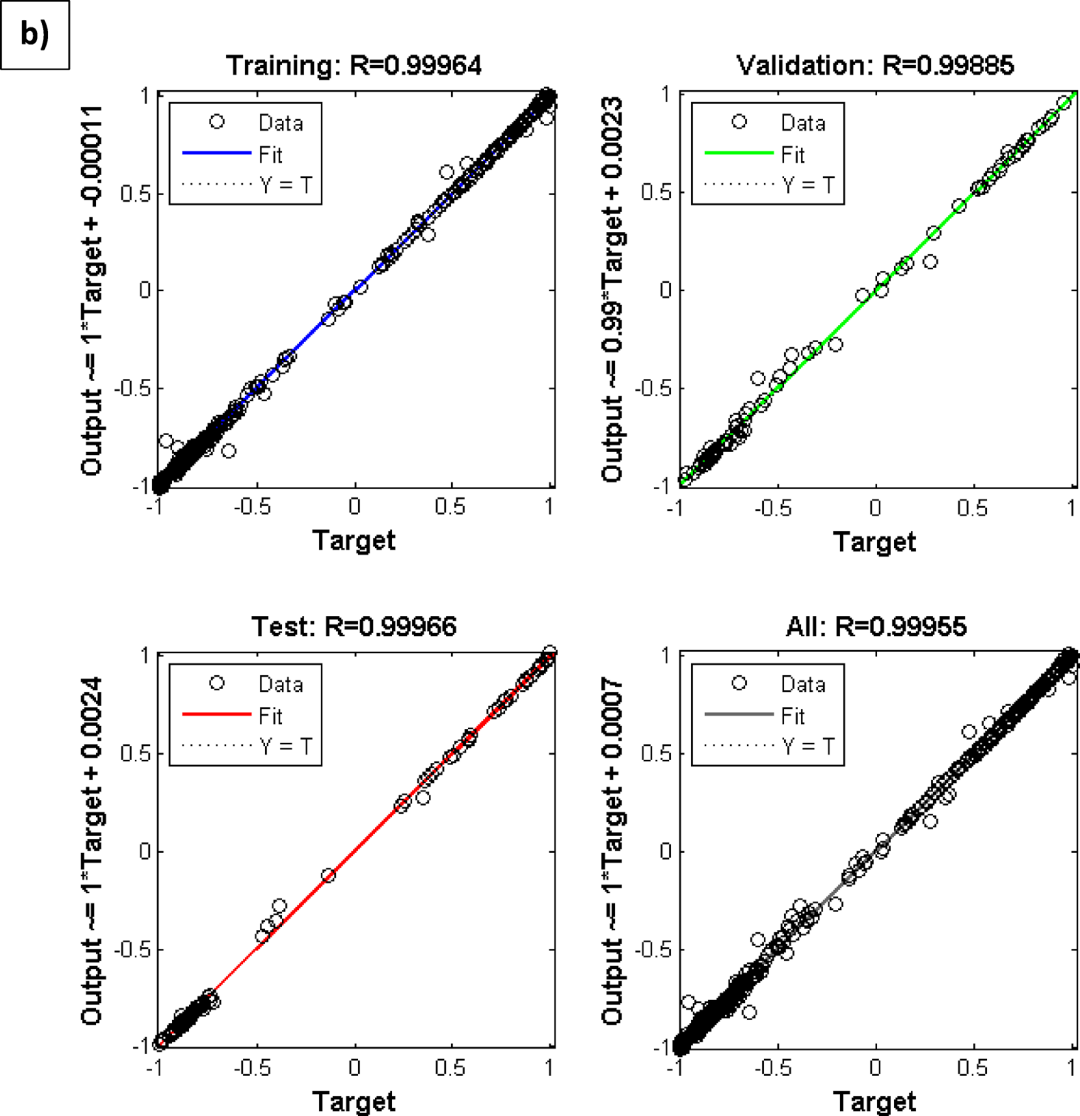
(**a**) the MSE values vs network iteration and (**b**) regression of data points using the ANN + GC model.

In Figure 2a and b, the MSE vs network iterations (epochs) and the regression of data points using the ANN+GC approach have been depicted.

The best MSE value has been obtained 2.06 × 10^-6^ at 3000 iterations, and the R^2^ value has been obtained about 0.999. In Figure 2b, the training, testing, and validation of the proposed ANN+GC model have been depicted. As shown in Figure 2b, the model can correlate all data points satisfactory.

**Fig. 3:**
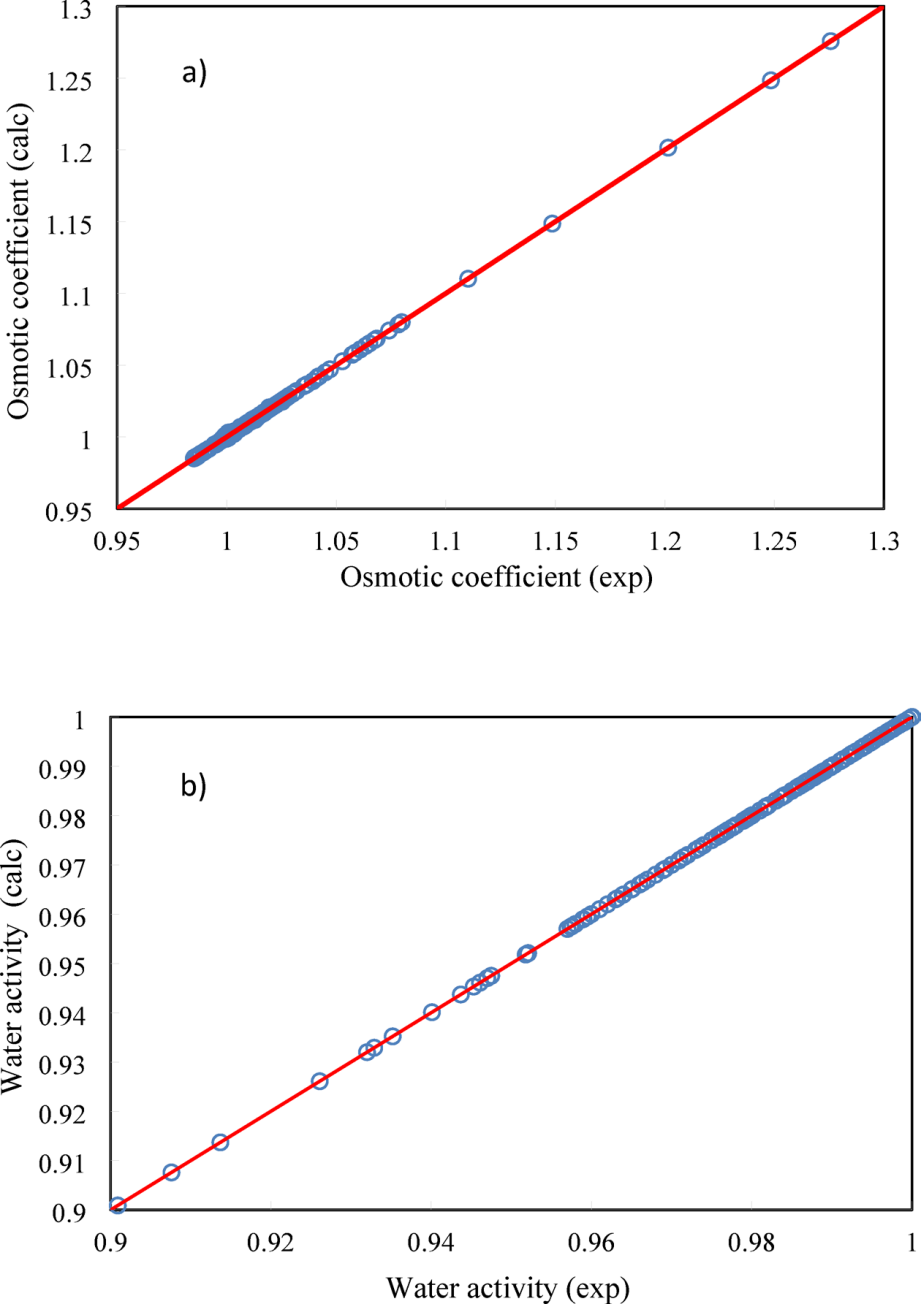

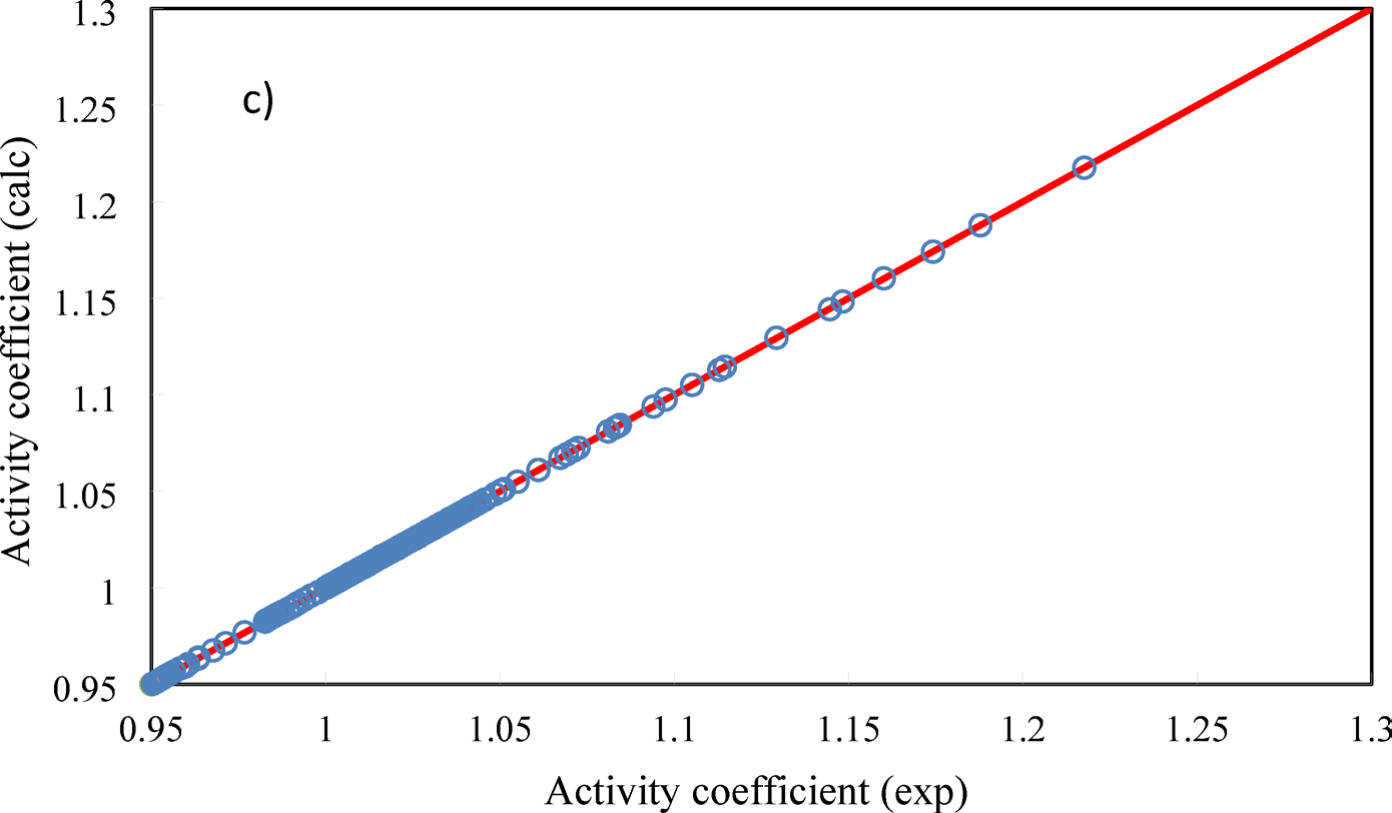
Experimental vs correlated data using ANN approach. (**a**) osmotic coefficient, (**b**) water activity, and (**c**) sugars activity coefficient.

In Figure 3, osmotic coefficient, water activity, and activity coefficient of ANN model have been compared to the experimental data.

The results show that the ANN+GC approach can estimate the water activity, osmotic coefficient, and activity coefficient of sugars accurately. The trial and error should be used to identify the optimum ANN architecture. A proposed ANN architecture cannot reproduce the same results after each running of the network, exactly ^27^. Nevertheless, if the weight and Bias of input, hidden, and output layers are known, the ANN architecture can produce the same results after each run. As well, the matrix of neural network weights can be used to determine the relative importance of the various input variables on the output variable. In this regard, in Table 3 the weight and Bias values of hidden and output layers have been reported.

The determination of the importance of each input variable (sensitivity analysis of input variables) can be studied using the weight connection between the input layer-hidden layer and between the hidden layer-output layer; Table 3. Garson suggested an equation based on partitioning of connection weights for sensitivity analysis of input variables as follows ^28^:

17 $$IF_{j} = \frac{{\mathop \sum \nolimits_{m = 1}^{Nh} \left( {\left( {\frac{{\left| {w_{jm}^{ih} } \right|}}{{\mathop \sum \nolimits_{k = 1}^{Ni} \left| {w_{km}^{ih} } \right|}}} \right).w_{mn}^{ho} } \right)}}{{\mathop \sum \nolimits_{k = 1}^{Nh} \left\{ {\mathop \sum \nolimits_{m = 1}^{Nh} \left( {\left( {\frac{{\left| {w_{km}^{ih} } \right|}}{{\mathop \sum \nolimits_{k = 1}^{Ni} \left| {w_{km}^{ih} } \right|}}} \right).w_{mn}^{ho} } \right)} \right\}}}$$

where *IF*_*j*_ is the relative importance of the *j*th input variable on output variable; *N*_*i*_ and *N*_*h*_ refer to the number of input and hidden neurons, respectively. The superscripts *i*, *h* and *o* refer to input, hidden and output layers, respectively. The subscripts *k*, *m* and *n* refer to input, hidden and output layers, respectively. *w* is connection weights.

**Fig. 4:**
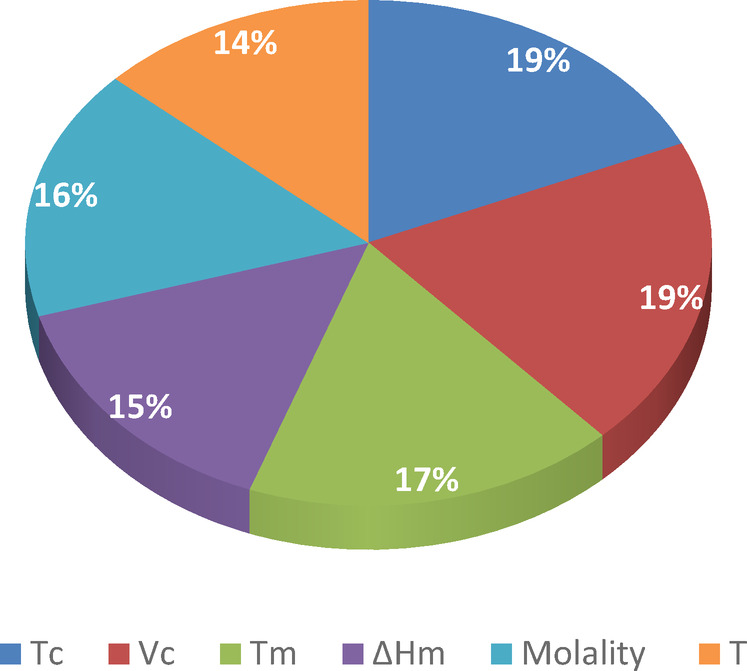
Relative importance (%) of input variables on the value of the osmotic coefficient, sugar activity coefficient, and water activity of aqueous sugar solutions.

The relative importance of input variables (IF_j_) have been calculated by Eq. (17), and depicted in Fig. 4.

It can be seen that logically all selected input variables have strong effects on the osmotic coefficient, activity coefficient, and water activity values with importance equal to 14% to 19 %. As shown in Fig. 4, the melting enthalpy and temperature have a minor effect on the aforementioned properties (IF = 14%). However, the relative importance of critical volume and critical temperature are the same values. Using a hybrid method based on a Feed Forward (FF)-ANN approach the osmotic coefficient, activity coefficient, and water activity of aqueous sugar solutions have been estimated up to high sugar concentrations.

### Thermodynamic modeling

In the case of sugar-water solutions, water parameters were adjusted using the vapor pressure and saturated liquid density experimental data. Five model parameters containing segment number *r*, segment diameter *σ*, segment-segment interaction energy *ε*, association energy ${\varepsilon }^{AB}$ and association volume ${\kappa }^{AB}$ have been adjusted for an associative component such as water. In this work, two association sites (one donor and one acceptor) have been considered for each water molecule. As well, the sugar molecules have been modeled as associative molecules. Number of association sites have been defined based on the OH group of molecules. For each OH group two association sites (one donor and one acceptor) have been considered (refer to Table 3). The osmotic coefficient experimental data in binary sugar-water system has been utilized to optimized the sugars parameters. The Eq. (18) has been used to optimize the model parameters:

18 $$OF=\sum_{i=1}^{N}\frac{\left|{\phi }_{i}^{exp}-{\phi }_{i}^{calc}\right|}{{\phi }_{i}^{exp}}$$

where *i* is data points, and *N* refers to total number of experimental data ^29^. In Table 4 the model parameters have been reported.

**Table 4:** Adjusted PHSC parameters of pure sugars and water.

Sugars	Parameters						T(K)	Max molality	Exp. data	no. of exp. data
	*r*(-)	$\sigma (\dot{\text{A}})$	$\varepsilon /{k}_{B}(\text{K})$	${\varepsilon }^{ab}/{k}_{B}(\text{K})$	${k}^{AB}$	N^Assoc^				
D-Glucose	7.919	3.240	338.532	2233.411	0.0701	10	303.08	4.8	^29^	24
Xylose	6.851	3.551	389.686	2268.548	0.055	8	308.15	3.0		20
L-Arabinose	8.078	2.121	194.955	2993.764	0.012	10	308.15	3.3		18
Xylitol	6.096	3.342	373.589	1526.725	0.032	10	308.15	3.3		19
D-Ribose	6.208	3.806	415.485	1572.824	0.057	10	308.15	3.3		20
D-galactose	7.911	2.969	418.363	2633.597	0.093	10	308.15	2.6		18
D-fructose	7.836	3.245	330.953	1495.777	0.068	10	308.15	5.5		21
D-mannose	9.175	2.445	336.939	2416.650	0.097	10	308.15	0.62		12
Sucrose	9.269	3.396	376.442	1414.893	0.017	16	303.15	3.9		22
Sorbitol	6.039	3.514	401.366	1522.868	0.073	12	308.15	3.6		21
Raffinose	9.939	3.735	433.741	1324.792	0.012	20	308.15	0.62		8
Maltitol	6.245	4.000	457.875	1055.046	0.058	12	308.15	3.6		14
Maltose	5.382	3.977	471.902	1782.704	0.046	16	308.15	3.6		16
Water	1.405	3.043	460.520	1633.280	0.0380	2	278–640	-	^30^	50

The water activity and activity coefficient of sugars have been predicated using the adjusted parameters in Table 4. The sugar model parameters, number of association sites, maximum molality, number of data points, and temperature have been presented in Table 4.

**Fig. 5:**
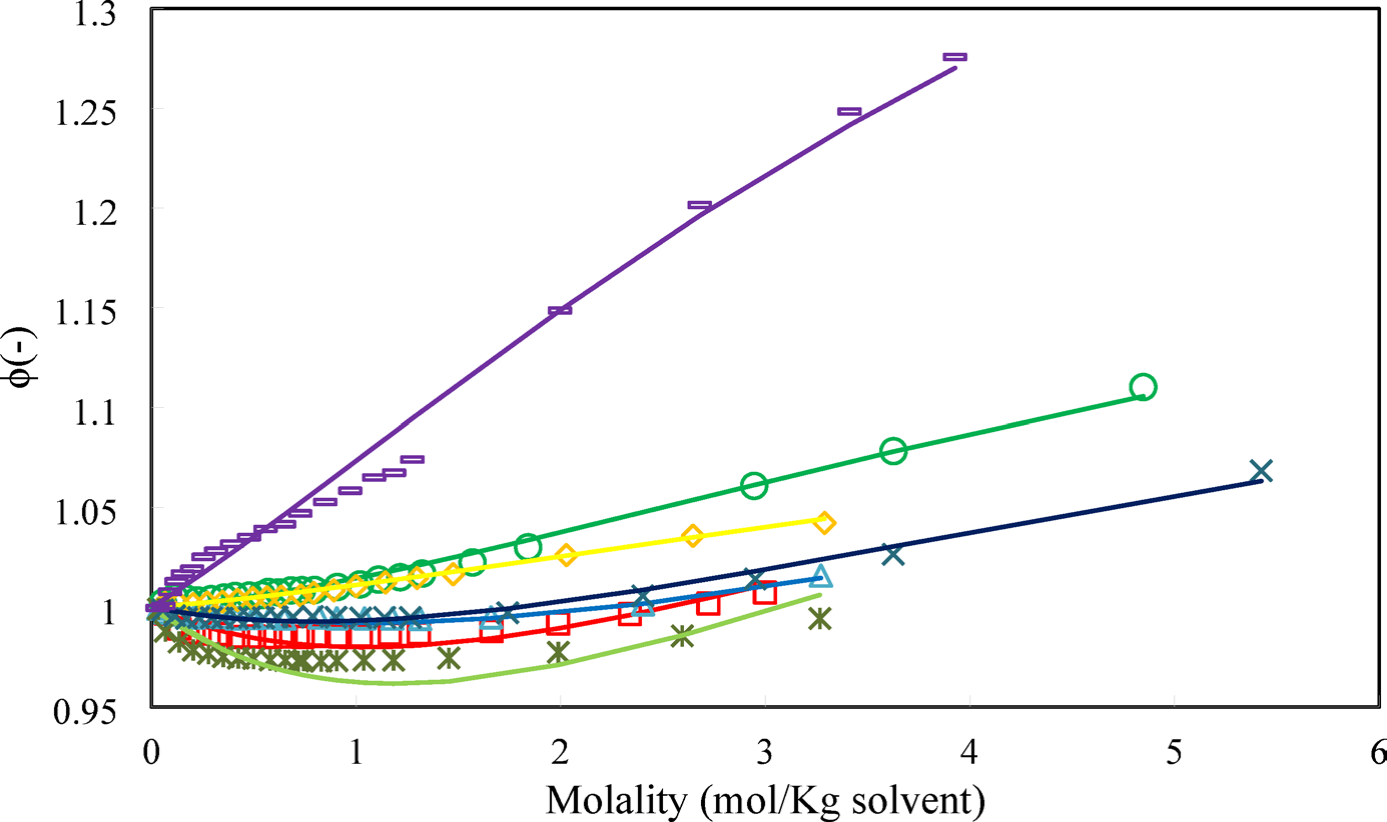
Osmotic coefficient of (○) D-Glucose, (□) Xylose, (∆) L-Arabinose, (◊) Xylitol, (*) D-Ribose, (⨯) D-fructose, (-) Sucrose. Lines refer to model calculations and symbols refer to experimental data ^29^ at 298.15 K.

**Fig. 6:**
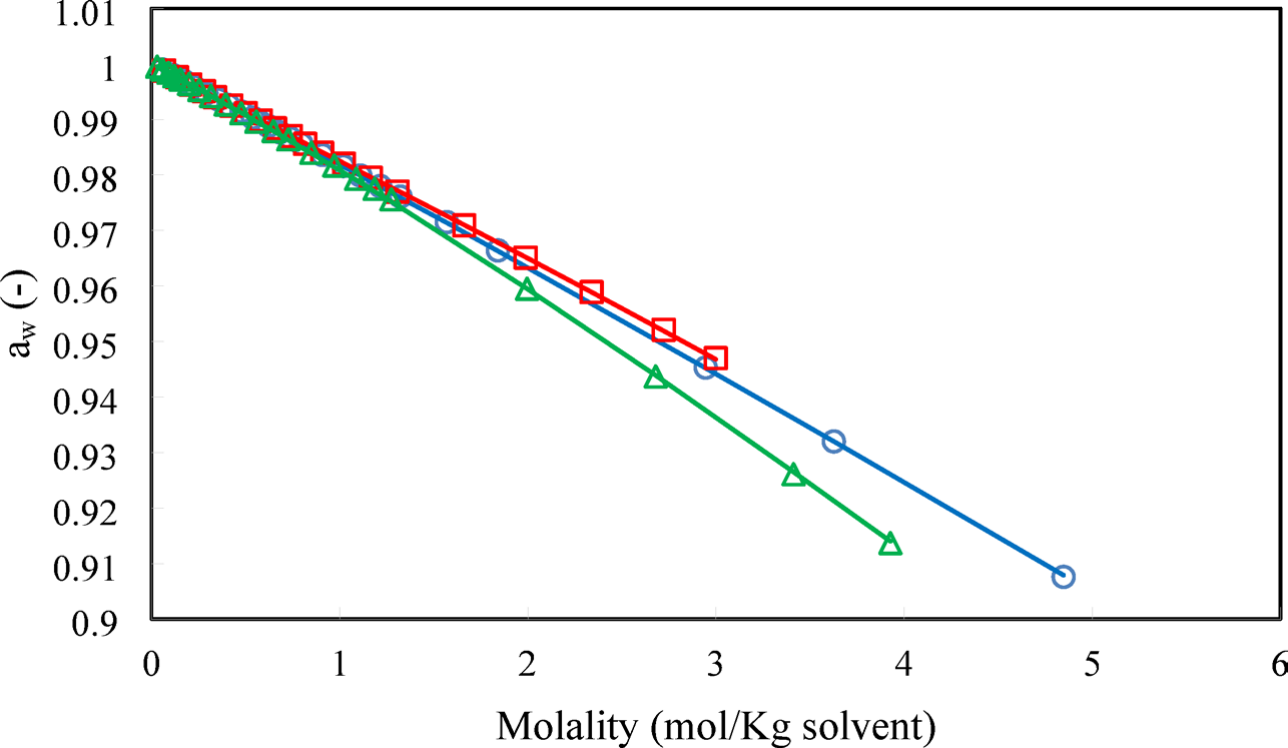
Water activity (○) D-Glucose, (□) Xylose, (∆) Sucrose. Lines refer to model calculations and symbols refer to experimental data ^29^ at 298.15 K.

**Fig. 7:**
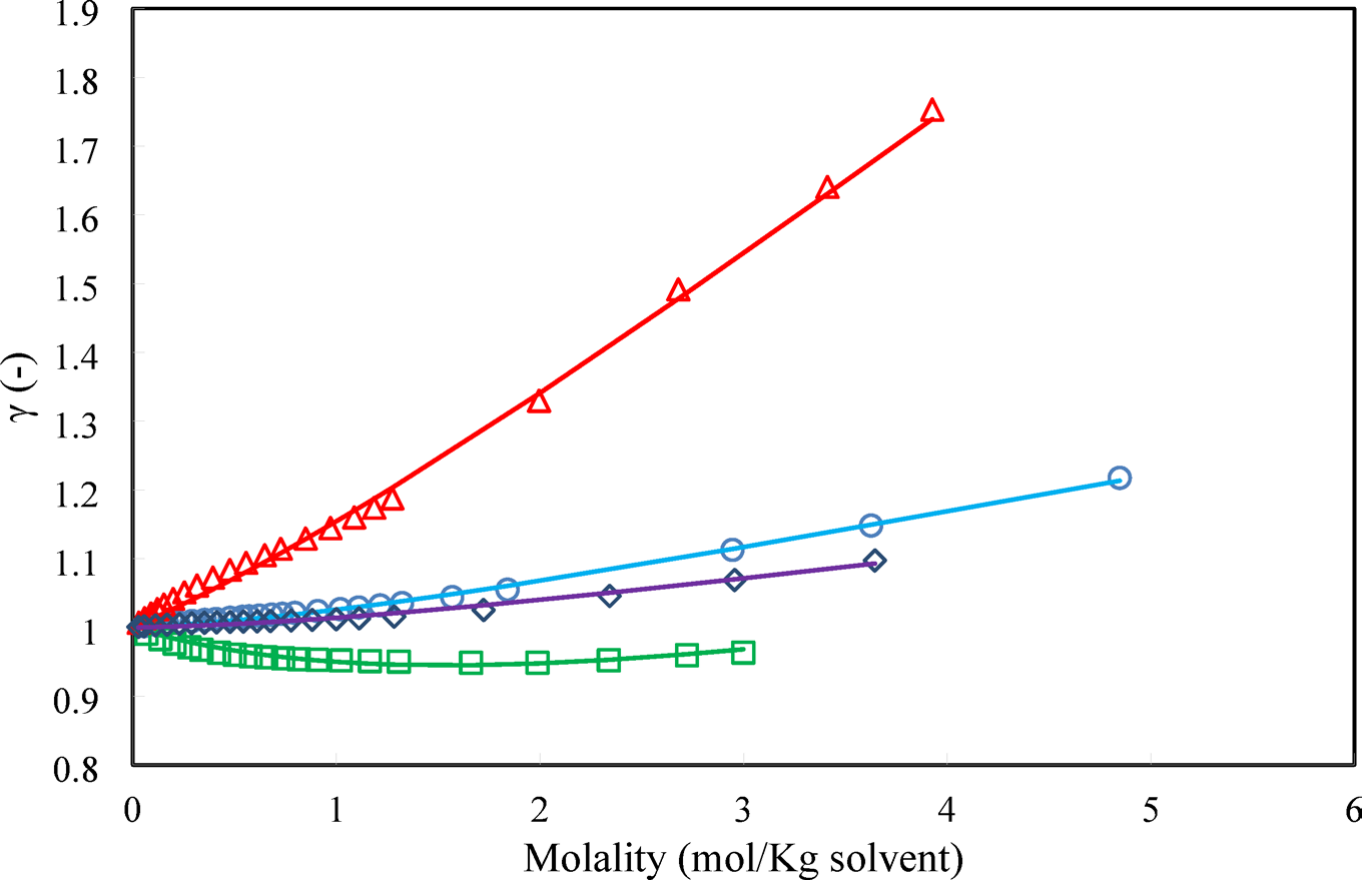
Activity coefficient (○) D-Glucose, (□) Xylose, (∆) Sucrose, (◊) Sorbitol. Lines refer to model calculations and symbols refer to experimental data ^29^ at 298.15 K.

In Figs. 5, 6 and 7, the model results have been compared to osmotic coefficient, water activity, and activity coefficient experimental data.

As shown in Figs. 5, 6 and 7, the PHSC EoS estimates the activity coefficient, water activity, and osmotic coefficient of binary sugar-water systems accurately.

As shown in Fig. 6, the activity of water decreases as the molality of sugar in water increases because the presence of sugar molecules disrupts the water’s structure and reduces the concentration of free water molecules available to participate in reactions. Pure water has an activity of 1.0. Solutions have activities less than 1.0. When you dissolve sugar in water, the sugar molecules (sucrose, for example) also form hydrogen bonds with water molecules. The sugar molecules essentially “tie up” some of the water molecules, reducing the number of water molecules that are free to interact with each other and exert their normal vapor pressure. The sugar molecules physically obstruct the water-water interactions. Therefore, adding sugar to water decreases water activity because the sugar molecules interfere with the water-water hydrogen bonding network, reducing the number of water molecules effectively available.

As shown in Fig. 7, while adding sugar to water always decreases water activity, the activity coefficient of sugar typically increases with increasing molality. This is due to a combination of solute–solute repulsions, changes in solvation, and excluded volume effects, all leading to positive deviations from ideal behavior. The specific behavior depends on the sugar, concentration range, and temperature. It must be noted that the prediction of the activity coefficient of sugars in solvent plays a crucial role in solubility calculations. In this regard, the activity coefficient of carbohydrates in aqueous solutions must be predicted accurately. In the crystallization process for the purification of carbohydrates, the sugar activity coefficient is needed in a wide range of temperatures and sugar concentrations to estimate the solubility of sugars. The use of the PHSC EoS provides a robust framework for estimating the activity coefficients of sugar solutions. By carefully choosing parameters and validating against experimental data, reliable predictions can be obtained. In the next section the PHSC EoS results have been compared to the ANN + GC approach.

### Comparison between the PHSC and ANN + GC models

In the case ANN + GC method, the input variables and the connection weights between inputs-hidden layers and hidden-output layers can be used to precited the osmotic coefficient, water activity, and activity coefficient of sugars over a wide range of sugar concentrations. On the other hand, the PHSC parameters (Table 3) can be used to predict the activity coefficient and water activity of sugars. In Table 5, the ARD% values of PHSC EoS and the ANN + GC approach have been reported.

**Table 5:** The ARD values of osmotic coefficient, water activity, and activity coefficient of sugars obtained by the PHSC EoS and ANN + GC models.

Sugars	ARD (%)						
	PHSC			ANN + GC			
	$\phi$	${a}_{w}$	$\gamma$	Dataset	$\phi$	${a}_{w}$	$\gamma$
D-Glucose	0.345	0.163	0.619	Correlated	0.0215	0.0018	0.0033
Xylose	0.340	0.007	0.404	Correlated	0.0105	0.0021	0.0041
L-Arabinose	0.124	0.002	0.160	Predicted	4.588	1.688	1.825
Xylitol	0.081	0.003	0.106	Correlated	0.0360	0.0025	0.0068
D-Ribose	0.663	0.011	0.767	Predicted	4.621	0.938	4.826
D-galactose	0.133	0.002	0.219	Correlated	0.062	0.0074	0.0076
D-fructose	0.162	0.007	0.154	Correlated	0.0562	0.0063	0.0085
D-mannose	0.984	1.235	1.578	Predicted	5.805	3.900	5.107
Sucrose	1.134	0.079	2.419	Correlated	0.0631	0.0022	0.0066
Sorbitol	0.165	0.005	0.155	Predicted	5.321	1.952	3.145
Raffinose	0.677	0.005	1.109	Correlated	0.0985	0.0039	0.0058
Maltitol	0.456	0.010	0.429	Correlated	0.0298	0.0045	0.0096
Maltose	0.399	0.012	0.425	Predicted	6.108	2.996	6.518
Average err	0.436	0.119	0.657		2.063	0.885	1.652

As shown in Table 5, the average ARD% values of the osmotic coefficient, water activity, and activity coefficient of the PHSC EoS have been obtained 0.436%, 0.119%, and 0.657%, respectively.

In the case of the ANN + GC approach, the prediction capability of the ANN + GC model has been investigated. In this regard, the osmotic coefficient, water activity, and sugar activity coefficient of five sugars containing L-Arabinose, D-Ribose, D-mannose, Sorbitol, and Maltose have been predicted using the ANN + GC model. It should be noted that the experimental data for the aforementioned sugars were not used in the development of the ANN. The network architecture, Bias, and weights of all layers were saved in the “saved network” program code; refer to Supplementary material. Six inputs of the aforementioned sugars were fed to the saved file to predict their osmotic coefficient, sugar activity coefficient, and water activity. Using the “saved network” and six input variables, the osmotic coefficient, sugar activity coefficient, and water activity were predicted satisfactory. The average ARD% values of the osmotic coefficient, water activity, and sugar activity coefficient have been obtained 5.3%, 2.3%, and 4.2%, respectively. In the Supplementary material, the complete MATLAB codes which include all the source codes of the programming have been provided.

**Fig. 8:**
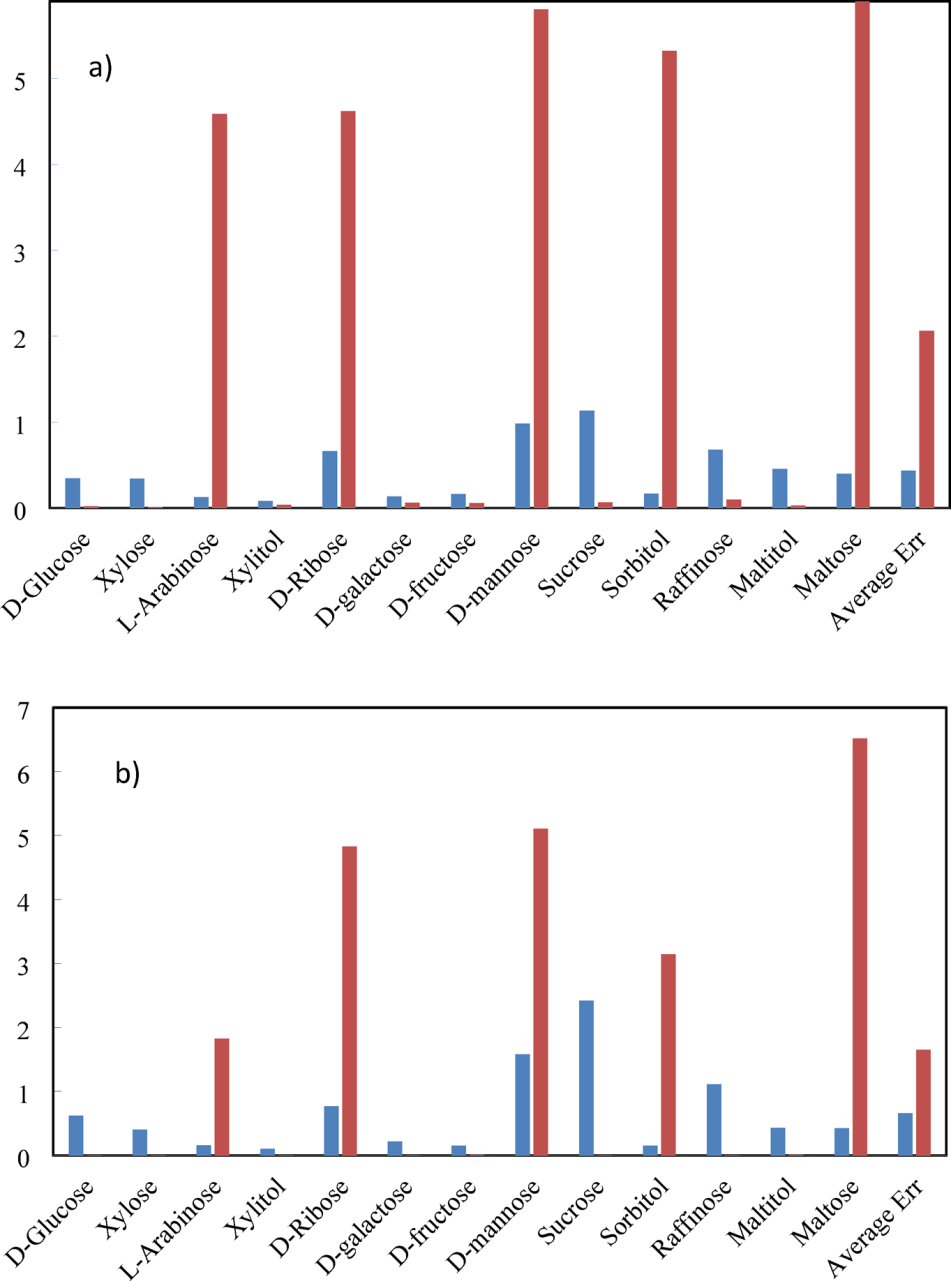
ARD% values of sugars osmotic coefficient (**a**) and activity coefficient (**b**). The PHSC model (blue), and ANN + GC model (Red).

In Fig. 8, the ARD% values of sugars osmotic coefficient, and activity coefficient of the PHSC EoS and ANN + GC approach have been compared.

As shown in Fig. 8, the average ARD% value of the osmotic coefficient and activity coefficient predicted by ANN + GC approach is higher than the PHSC model. It is due to the ARD% values of five sugars containing L-Arabinose, D-Ribose, D-mannose, Sorbitol, and Maltose. The reported ARD% values of the aforementioned sugars are pure predictions. Because their experimental data was not utilized for network development. After training, ANNs can provide rapid predictions, significantly speeding up the modeling process compared to EoS-based approaches. This is the main benefit of the ANN approaches compared to the thermodynamic models such as the PHSC EoS.

The results show that the proposed ANN architecture can predict the osmotic coefficient, water activity, and activity coefficient of aqueous sugar solutions using the universal weight and Bias connections of input, hidden, and output layers. The proposed methodology for the estimation of critical properties using the GC methods helps us to use the ANN approach for the new-designed sugars.

### Solubility of sugars in water

In this section, the solubility of several sugars in water has been investigated using the ANN + GC and the PHSC EoS. In this regard, the obtained solute activity coefficient must be used to calculate the sugar solubility. The Solid–Liquid Equilibrium (SLE) phase-equilibrium conditions between the liquid and the solid phase are applied to estimate the solubility of sugars in liquid solvents.

The mole fraction of component *i* in a solvent at equilibrium (solubility) is calculated as follows:

19 $$- \ln \left( {x_{i} \gamma_{i}^{solute } } \right) = \frac{{\Delta H_{m} }}{R}\left( {\frac{1}{T} - \frac{1}{{T_{m} }}} \right) + \frac{{\Delta C_{p} }}{R}\ln \left( {\frac{{T_{m} }}{T}} \right) - \frac{{\Delta C_{p} }}{R}\left( {\frac{{T_{m} }}{T} - 1} \right)$$

where *x*_*i*_ and ${\gamma }_{i}^{solute}$ are the mole fraction and the symmetric activity coefficient of the sugars, respectively. *∆H*_*m*_ stands for the melting enthalpy, *T*_*m*_ is the melting temperature of the sugar and *∆C*_*p*_ is the difference in solute heat capacity between liquid and solid at the melting point, *R* is the gas constant, and *T* is the temperature. The melting temperature, melting enthalpy, and heat capacity for six samples were reported in literature ^8,31,32^; see Table 6. In Table 6, the ARD% values of the PHSC and ANN + GC models have been reported.

**Table 6:** Melting temperature, melting enthalpy, heat capacity, and the ARD% values of carbohydrate solubility at 1 bar.

Sugars	T_m_ (K)	$\Delta C_{p} \,\left( {{\text{J}}/{\text{mol}}/{\text{K}}} \right)$	$\Delta {H}_{m}$ (J/mol)	ARD%		∆T(K)	Max molality	No. exp. data	Ref. exp. data
				PHSC	ANN				
D-Glucose	423.2	183.0	42,432	17.6	20.2	282–334	15	6	^33^
Xylose	416.2	97.3	29,939	6.7	8.4	298–348	25	6	^34^
Xylitol	367.5	77.7	33,678	9.1	6.3	293–327	33	8	^35^
D-galactose	438.1	153.0	43,740	8.6	18.2	298–348	8.5	6	^34^
D-fructose	378.2	99.2	26,029	5.6	4.7	270–313	30	6	^36^
Sucrose	459.1	261.0	46,187	7.5	9.2	278–333	22	8	^37^

**Fig. 9:**
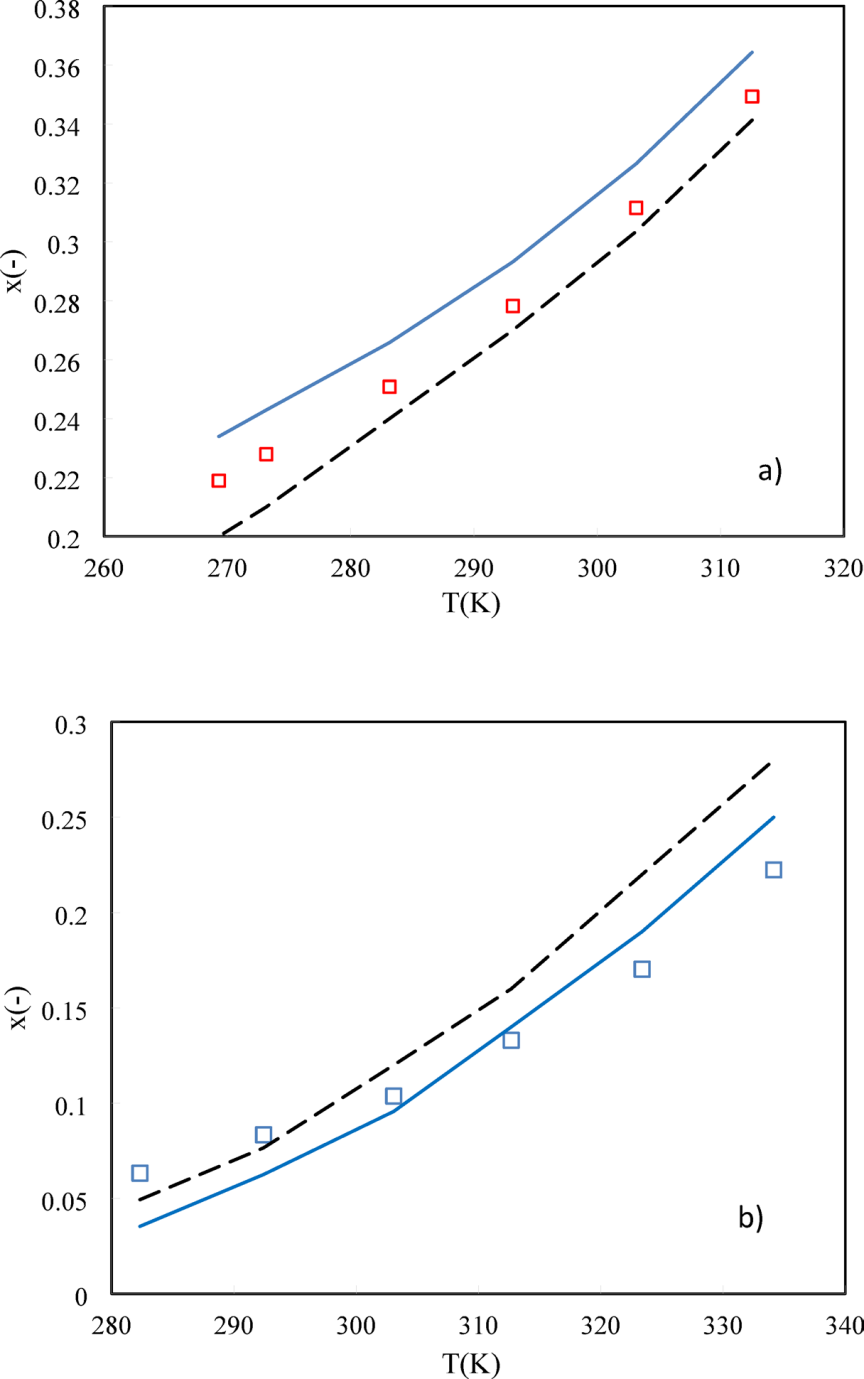

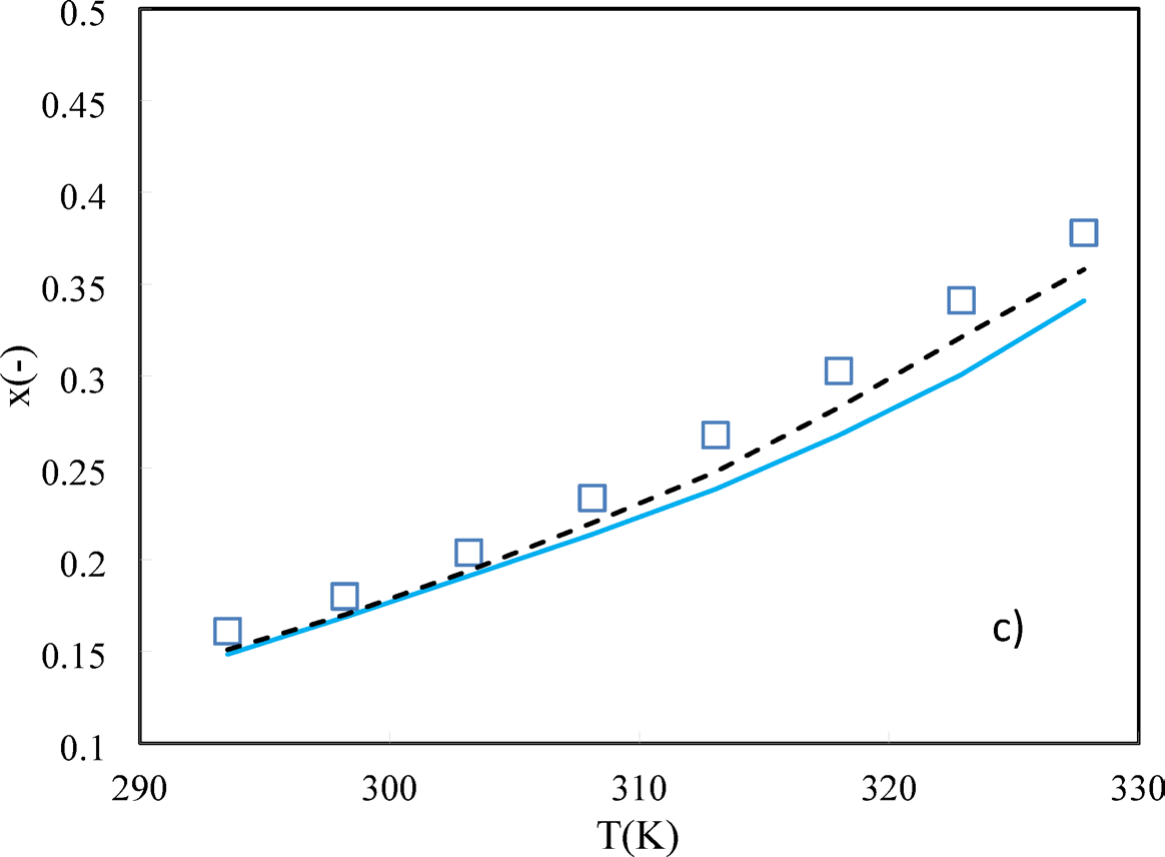
Solubility of (**a**) D-fructose, (**b**) D-Glucose, and (**c**) Xylitol in water. Solid line is PHSC and dashed line refers to ANN + GC approach. Symbols are experimental data ^33–36^.

As shown in Eq. (19), the solubility of sugars in water depends on the solute activity coefficient, melting temperature, $\Delta {C}_{p}$, and $\Delta {H}_{m}$. The activity coefficient of solute at desired molality can be estimated using ANN + GC and PHSC approaches in Sections “The PHSC EoS” and “The ANN models”. Therefore, the solubility of sugars in water can be predicted without using any additional adjustable parameters or experimental data. The average ARD% values of the PHSC and ANN + GC approach have been obtained about 9.2% and 11.2%, respectively. In the case of D-glucose and D-galactose, higher ARD% values were observed, especially in the ANN + GC method. In Fig. 9, the PHSC and ANN + GC results have been compared to experimental solubility data.

As shown in Fig. 9, the PHSC and ANN + GC models can predict the solubility of D-fructose, D-Glucose, and Xylitol in water satisfactory.

The results of this work show that the ANN and the perturbation-based EoS such as the PHSC model can be used to estimate the thermodynamic properties of aqueous sugar solutions up to high sugar concentration, satisfactory. The ANN + GC method is a robust model when experimental data is not available for the new-designed carbohydrates. In the ANN + GC method, the GC approach helps us to estimate the sugar properties using the molecular structure, then the ANN approach estimates the osmotic coefficient and sugar activity coefficient, satisfactory. On the other hand, the PHSC model can extend to high pressure (or high temperature), accurately. Also, the relationship between thermodynamic properties (first-order derivative thermodynamic properties such as density and second-order derivative thermodynamic properties such as speed of sound or heat capacity) helps us to estimate/predict other thermodynamic properties over a wide range of pressures and temperatures by using the PHSC model.

## Conclusion

In this work, the ANN + GC approach has been proposed to predict the osmotic coefficient, activity coefficient, and water activity of aqueous sugar solutions. The input layer of the ANN method has been estimated using the GC approach. Three output layers, and one hidden layer (with 32 neurons) have been considered to develop the ANN + GC method. The average ARD% values of the predicted osmotic coefficient, water activity, and activity coefficient have been obtained 5.3%, 2.3%, and 4.2%, respectively. Also, the PHSC EoS has been used to study the aforementioned thermodynamic properties. The PHSC EoS model parameters have been obtained using osmotic coefficient experimental data. Then the activity coefficient and water activity of sugar solutions have been predicted. The average ARD% values of the osmotic coefficient, water activity, and activity coefficient of the PHSC EoS have been obtained 0.436%, 0.119%, and 0.657%, respectively. The results show that the PHSC EoS and the ANN + GC approach can predict the activity coefficient, osmotic coefficient, and water activity of aqueous sugar solutions satisfactory. In the ANN + GC method, the GC approach helps us to estimate the sugar properties using the molecular structure. In the case of the PHSC EoS, the relationship between thermodynamic properties helps us to estimate/predict other thermodynamic properties over a wide range of pressures and temperatures. The solubility of six sugars in water was predicted to evaluate the performance of the ANN + GC and PHSC models. The results show that, the ANN + GC approach can predict the solubility of sugars in water in the absence of experimental data. By considering a fully predictive approach of the PHSC model, the results were in good agreement with experimental data. The proposed models can be extended for the calculation of thermo-physical properties of sugar-containing systems in future works.

## Electronic supplementary material

Below is the link to the electronic supplementary material.

Supplementary Material 1

## Data Availability

All data generated or analysed during this study are included in this published article [and its supplementary information files].
