# Supplementary material for: Prediction of thermodynamic properties of aqueous carbohydrates solution using the PHSC and ANN models
Source: Sci Rep. 2025 Jul 1;15:21539. doi: 10.1038/s41598-025-06552-2 (PMC12216867; doi:10.1038/s41598-025-06552-2)
Supplement: Supplementary file 1 — Supplementary Material 1 [file 41598_2025_6552_MOESM1_ESM.docx]

%Osmotic coefficient, activity coefcient, and water activity of

%Carbohydrate_ANN

%*********

%THIS IS THE MATLAB CODE FOR TRAINING AN ANN WITH osmotic and activity coeffcient DATA,

%USING AS INDEPENDENT

%TRAINING SECTION

%**********************

%Reading independent variables for training

p = xlsread('variables_cp_training_1');p=p';

% %Reading the dependent variable for training

t = xlsread('cp_for_training');t=t';

% Normalization of all data (values between -1 y +1)

[pn,minp,maxp,tn,mint,maxt] = premnmx(p,t);

% Definition of ANN:(topology, activation functions, training algorithm)

net=newff(minmax(pn),[5,32,3],{'tansig','tansig','purelin'},'trainlm');

% Definition of frequency of visualization of errors during training

net.trainParam.show = 10;

% Definition of number of maximum iterations (epochs) and global error between iterations (goal)

net.trainParam.epochs = 2000; net.trainParam.goal = 1e-7;

net.divideFcn='dividerand';

net.divideParam.trainRatio=0.7; %--- Train Ratio

net.divideParam.valRatio=0.15; %--- Validation Ratio

net.divideParam.testRatio=0.15; %--- Test Ratio

%Network starts: reference random weights and gains

w1 = net.IW{1,1}; w2 = net.LW{2,1}; w3 = net.LW{3,2};

b1 = net.b{1}; b2 = net.b{2}; b3 = net.b{3};

%First iteration with reference values and correlation coefficient

before_training = sim(net,pn);

corrbefore_training= corrcoef(before_training,tn);

%Training process and results

[net,tr]=train(net,pn,tn);

after_training = sim(net,pn);

% Back-Normalization of results, from values between -1 y +1 to real values

after_training = postmnmx(after_training,mint,maxt); after_training=after_training';

Res = sim(net,pn);

% Saving results, correlated densities in an Excel file

dlmwrite('cp_correlated.xls',after_training,char(9));

%Saving the nerwork (weigths and other files)

save w1_cp

%

%PREDICTION SECTION

%*****************************

%Reading weigth and other characteristics of the tained ANN saved in the file W

load w1_cp

% Reading of Excel file with new indepent variables to predict osmotic,

% activity coeffcient

pnew = xlsread('variables_cp_ prediction_1'); pnew=pnew';

% Normalization of all variable (values between -1 y +1)

pnewn = tramnmx(pnew,minp,maxp);

% Testing the ANN obtaining the properties for the variables provided by the file

% variables_cp_for_prediction

anewn = sim(net,pnewn);

% Transformation of the normalized exits (between -1 y +1) determined by the ANN to real values

anew = postmnmx(anewn,mint,maxt); anew=anew';

% Saving the predicted properties in en Excel file

dlmwrite('cp_ predicted.xls',anew,char(9));
